# The Value of the Correlation Coefficient Between the ICP Wave Amplitude and the Mean ICP Level (RAP) Combined With the Resistance to CSF Outflow (Rout) for Early Prediction of the Outcome Before Shunting in Posttraumatic Hydrocephalus

**DOI:** 10.3389/fneur.2022.881568

**Published:** 2022-04-26

**Authors:** Chao Zhang, Si-Yu Long, Wen-dong You, Xu-xu Xu, Guo-Yi Gao, Xiao-Feng Yang

**Affiliations:** ^1^Emergency and Trauma Center, The First Affiliated Hospital, Zhejiang University School of Medicine, Hangzhou, China; ^2^Department of Nursing, Ren Ji Hospital, School of Medicine, Shanghai Jiao Tong University, Shanghai, China; ^3^Department of Neurosurgery, Minhang Hospital, Fudan University School of Medicine, Shanghai, China; ^4^Department of Neurosurgery, Shanghai General Hospital, Shanghai Jiao Tong University School of Medicine, Shanghai, China

**Keywords:** resistance to CSF outflow, CSF infusion test, RAP, hydrocephalus, traumatic brain injury, pressure-volume compensation, shunting

## Abstract

**Objective:**

To evaluate the value of the correlation coefficient between the ICP wave amplitude and the mean ICP level (RAP) and the resistance to CSF outflow (Rout) in predicting the outcome of patients with post-traumatic hydrocephalus (PTH) selected for shunting.

**Materials and Methods:**

As a training set, a total of 191 patients with PTH treated with VP shunting were retrospectively analyzed to evaluate the potential predictive value of Rout, collected from pre-therapeutic CSF infusion test, for a desirable recovery level (dRL), standing for the modified rankin scale (mRS) of 0–2. Eventually, there were 70 patients with PTH prospectively included as a validation set to evaluate the value of Rout-combined RAP as a predictor of dRL. We calculated Rout from a CSF infusion test and collected RAP during continuous external lumbar drainage (ELD). Maximum RAP (RAPmax) and its changes relative to the baseline (ΔRAPmax%) served as specific parameters of evaluation.

**Results:**

In the training set, Rout was proved to be a significant predictor of dRL to shunting, with the area under the curve (AUC) of 0.686 (*p* < 0.001) in receiver-operating characteristic (ROC) analysis. In the validation set, Rout alone did not present a significant value in the prediction of desirable recovery level (dRL). ΔRAPmax% after 1st or 2nd day of ELD both showed significance in predicting of dRL to shunting with the AUC of 0.773 (*p* < 0.001) and 0.786 (*p* < 0.001), respectively. Significantly, Rout increased the value of ΔRAPmax% in the prediction of dRL with the AUC of 0.879 (*p* < 0.001), combining with ΔRAPmax% after the 1st and 2nd days of ELD. RAPmax after the 1st and 2nd days of ELD showed a remarkable predictive value for non-dRL (Levels 3-6 in Modified Rankin Scale) with the AUC of 0.891 (*p* < 0.001) and 0.746 (*p* < 0.001).

**Conclusion:**

Both RAP and Rout can predict desirable recovery level (dRL) to shunting in patients with PTH in the early phases of treatment. A RAP-combined Rout is a better dRL predictor for a good outcome to shunting. These findings help the neurosurgeon predict the probability of dRL and facilitate the optimization of the individual treatment plan in the event of ineffective or unessential shunting.

## Introduction

Posttraumatic hydrocephalus (PTH) is an excess accumulation of intracranial cerebrospinal fluid (CSF), following traumatic brain injury (TBI). PTH has been reported to prolong hospital stay and treatment costs and is associated with an increased risk of unfavorable outcomes, following TBI (1, 2). Although the incidence of PTH in patients suffering from TBI has been reported to be as high as 31.6% in recent research (3); PTH is still potentially under-diagnosed and under-treated.

Shunting is regarded as an effective treatment and has been widely used in curing idiopathic normal pressure hydrocephalus (iNPH) (4, 5). Since the mechanism of injury leading to PTH may be complex, involving disruption of normal CSF circulation by TBI-related intraventricular hemorrhage (IVH), space-occupying lesions, or alteration of CSF flow dynamics due to disturbance of CSF reabsorption (6–8). Therefore, there is an urgent need for creating a more efficient diagnostic tool to screen out suitable patients with PTH for shunting and optimize individual patients' treatment in case of ineffective and unnecessary surgery.

The resistance to CSF outflow (Rout), originated from the rise of ICP during infusion test compared to the baseline ICP, has been applied and trialed in iNPH and NPH of mixed pathogeny to guide shunting decisions, but it has not been investigated sufficiently in PTH cohorts for this intension. There is no unified threshold for Rout in predicting the outcome of shunting. The way to the application of Rout as a diagnostic and outcome prediction tool was laid early on with research from Børgesen et al. (9), who reported a >80% post–shunt placement success rate for patients with Rout ≥ 12.5 mmHg·min/ml. The milestone Dutch NPH trial (10) provided the necessary evidence for the use of the most common threshold for Rout, 18 mm Hg·min/ml, reporting a 92% success rate. This study pioneered quantitatively reporting clinical investigations for the patients before and after shunting; however, those highly positive results have never since been replicated (11). The current knowledge about Rout and the different proposed thresholds for diagnosis in NPH/iNPH are summarized in Table 1. Czosnyka et al. (14) improved the constant infusion method and developed it into a computerized set-up more suited for modern clinical application (15). The recommended threshold for shunting is between 12 and 18 mmHg/ml/min with the constant infusion method (9, 10, 12, 16). Another methodological problem is that a patient with hydrocephalus in an irreversible phase of the disease can have apathological resistance but not with shunt responsiveness, reducing the sensitivity of the test for a predictive value. Rout alone as a predictive tool for predicting the desirable recovery level (dRL) to shunting is not convincing enough.

**Table 1:** Studies of Rout and RAP: prediction of an outcome after CSF shunting.

**References**	**Study design**	**Participants (number)**	**Purpose**	**Variables studied**	**Findings regarding prognosis or treatment**
Børgesen et al. (9)	Prospective study	183	To evaluate the function of Rout in predicting the prognosis in patients with hydrocephalus	Rout	Rout ≥ 12 mmHg min/ml, with NA PPV and 100% NPV
Boon et al. (10)	Multicenter prospective study	101	Examined whether measurement of Rout predicts outcome after shunting for patients with normal-pressure hydrocephalus	Rout, Evans' index; ventricular index	Rout ≥ 12 mmHg min/ml, with 81% PPV and 50% NPV; Rout ≥ 18 mmHg min/ml, with 92% PPV and 34% NPV; highest likelihood ratio of 3.5 for Rout = 18 mmHg min/ml
Kahlon et al. (40)	Single-center retrospective cohort	68	To compare the Rout and CSF tap test for predicting the outcome of shunt surgery in NPH patients	Rout, mRS score	Rout ≥ 14 mmHg min/ml, with 80% PPV and NANPV; Strong correlation between Rout and outcome
Wikkelsø et al. (41)	Multicenter prospective study	115	To determine the predictive values of the CSF Tap Test and Rout for the outcome of shunting in Inph.	Rout, Evans' index, Mini-Mental State Examination scores	Rout ≥ 12 mmHg min/ml, with 86% PPV and 18% NPV; Rout ≥ 18 mmHg min/ml, with 94% PPV and 18% NPV; No correlation between Rout and outcome
Nabbanja et al. (12)	Prospective study	310	To study patients with an iNPH to compare the parameters and clinical improvement after shunting	Rout, ICP, RAP, AMP	Both Rout ≥ 12 and 18 mmHg min/ml are the best thresholds for estimation; ICP, RAP, and AMP are poorly related to outcome
Schuhmann et al. (13)	Single-center prospective study	32	To investigate the diagnostic potential of overnight ICP monitoring in shunted patients	ICP, RAP, AMP	ICP, AMP, and RAP can benefit the assessment of shunt function. RAP > 0.6 indicates shunt malfunction or hydrocephalus
Cynthia Mahr et al. (42)	Retrospective cohort	74	To analyze the diagnostic and predictive values of clinical tests, CSF dynamics, and intracranial pulsatility tests, compared with ELD	Rout, RAP, mRS score	Rout > 14 mmHg min/ml +RAP > 0.8 in iNPH, with 60% PPV and 65% NPV. Median RAP was not significantly different in iNPH

*NA, not available; NPV, negative predictive value; PPV, positive predictive value; NPH, normal-pressure hydrocephalus; iNPH, idiopathic NPH; AMP, pulse amplitude of ICP; ELD, external lumbar drainage; mRS, modified Rankin Scale*.

The correlation coefficient between the ICP wave amplitude and the mean ICP level (RAP) was originally designed as a potential descriptor of neurological deterioration in patients with TBI by Czosnyka et al. (17). Schuhmann et al. (13) suggested that RAP > 0.6 indicates shunt malfunction or hydrocephalus. With the ability to effectively demonstrate how the CSF compensatory reserve changes over time, RAP has been gradually introduced as a nearly response assessment parameter to differentiate responders from non-responders early in hydrocephalus according to its relative dynamic variations (13, 18, 19). The current knowledge about RAP and the different proposed thresholds for diagnosis in NPH/iNPH are summarized in Table 1.

Both Rout and RAP are potential indices to assess the probable shunt function of patients with PTH. We measured parameters, such as Rout, RAP, and calculated RAP-related parameters, to evaluate their predictive value of an outcome with patients with PTH after shunting. Furthermore, whether the combination of two indices will enhance the predictive power for dRL to shunting is unclear. To our knowledge, nobody has explored the approach before. Therefore, in our study, we firstly explored the predictive value of pre-therapeutic Rout for dRL, confirmed the predictive significance of Rout and RAP-related parameters, and explored the predictive value of combining them.

## Methods

### Patients

As a training set, 191 patients with PTH were treated with an infusion test, followed by ventriculoperitoneal shunting (VP) or lumboperitoneal shunting (LP) (consultant decisions based on clinical, radiological assessment and the infusion results) in our institution (Department of Neurosurgery, The First Affiliated Hospital, Zhejiang University) from August 2015 to July 2018. The evaluable pre-therapeutic Rout and the level of modified Rankin Scale (mRS) after shunting were retrospectively analyzed. These were all patients with ventriculomegaly on CT or MRI (as reported by an experienced consultant radiologist) and with clinical characteristics of PTH.

To confirm the predictive value of Rout and RAP, and to explore the predictive possibility of combining them, patients aged between 16 and 72 years with diagnosed PTH within 2 months were consecutively and prospectively enrolled in our study as a validation data set, from October 2018 to February 2020. Our specific inclusion criteria were as follows:

1. In our study, all patients with PTH should suffer from a clinical status with a level running from 3 to 6 in modified Rankin Scale (mRS) before enrolling, because we need to find the potential patients who may benefit from shunting and to reduce the baseline error, which can disturb the outcome assessment based on mRS. Then, they, firstly, underwent an infusion test to generate Rout data. Secondly, they received continuous lumbar drainage to collect RAP for consecutive 48 h. Finally, shunt operation without surgical taboos was implemented with them based on clinical, radiological assessments and the test results.
2. No removed bone flap at the time of tests, because decompressive craniectomy (DC) has significant effects on pressure-volume compensation (20, 21). If DC had been previously implemented, a cranioplasty should have been accomplished 4 weeks or more before the tests to enable restoration of the intracranial circulation (CSF as well as cerebral blood flow) (22, 23).
3. Computed tomography or brain magnetic resonance imaging results, indicating communicating hydrocephalus with an Evans index ≥ 0.3 (24).
4. Baseline ICP < 15 mmHg because it is too hazardous for these patients with high ICP to undergo infusion tests or lumbar cistern drainage in case of cerebral hernia. And altered CSF dynamics should also be concerned (23).
5. Rout > 6 mmHg/min/ml without a probable high degree of atrophy from the CT/MRI as reported by a radiologist.

The exclusion criteria should include epilepsy, inflammation, non-communicating hydrocephalus, and other serious diseases, which make patients unable to tolerate invasive diagnosis and chemotherapy. Patients with acute hydrocephalus or intracranial hypertension, who required mannitol or glycerol fructose to reduce intracranial pressure, were also excluded. The patients taking recent antiepileptic drugs, such as sodium valproate and carbamazepine, were also excluded. Finally, normal controls were not available, as all studies in our center are performed on clinical indications.

The study was approved by the Ethical Committee of The First Affiliated Hospital attached to Zhejiang University School of Medicine. All the patients in the validation data set were provided with written informed consent.

### Infusion Test

Infusion studies were performed *via* lumbar puncture with the patient in the lateral recumbent position. An 18-G spinal needle was performed below L3. The patient's head was carefully positioned to align the inion with the spine horizontally and parallel to the floor. The needle was connected through a 3-way tap, with a pressure-recording device to one side and with an infusion pump to the other side (pump, Grase by 3100; recorder, Codman, Medtronic, USA). The baseline steady state of CSF pressure was recorded for at least 10 min (range, 10–20 min). To avoid systematical error, we were more inclined to take constant-rate infusion other than bolus injection. Once the intracranial pressure (ICP) reading remained steady, infusion with saline solution was started at a constant rate of 1.5 ml/minute in all patients, and recording was continued for a further 10 min after ICP had reached its plateau but ≤ 15 mmHg. The CSF pressure was recorded continuously for 15–35 min (mean, 25 min). In addition, the infusion was terminated either if CSF pressure increased continuously until reaching a Ro > 18 mmHg/ml/minute or once a steady-state plateau pressure was approached (25). Then, the CSF Rout was calculated according to the equation described by Czosnyka et al. (14).

### Continuous Lumbar Drainage Test

As the infusion test is accomplished, the next day, a standard lumbar puncture was performed, usually in the lumbar 4–5 interspinous space, with a large-bore Tuohy needle (a 14–16 gauge). As the CSF was encountered, the curve of the needle was directed superiorly, the stylet was removed, and the catheter was advanced into the subarachnoid space for about 20–22 cm. Then, a (a 17–18 gauge) catheter (Medtronic, USA) was slowly inserted with one hand, while the needle was simultaneously extracted. The drain was attached to a three-way tap with a pressure-recording device (Codman, Medtronic, USA) to one side and with an external drainage sterile container to the other side. The catheter was then taped over the patient's flank. The lumbar drain was fixed to the shoulder level, and the patients were advised to have complete bed rest. We paid close attention and monitored the drain to avoid overtraining. Once the monitored ICP had reached its plateau but ≤15 mmHg, we started to open drainage. The lumbar drain was set to drain 10–15 cc per hour and approximately 300–400 cc in 2 days. Data were processed using Neumatic DCR + software (Haoju, Shanghai, China) and saved in our Neumaticstudy database.

### Outcome Assessment

Modified Rankin Scale (mRS) has remarkable strengths in evaluating the outcome of spontaneous subarachnoid hemorrhage (SAH) and hydrocephalus (26, 27). More importantly, mRS covers the degrees of gait and defecation disorder, which are two important clinical criteria for hydrocephalus assessment. Therefore, we decided to use mRS as an indicator to judge the prognosis instead of Glasgow Outcome Scale (GOSE). MRS is a 7-level outcome scale, running from perfect health without symptoms to death, which shows as follows:

0: no Symptoms.
1: no significant disability—able to carry out all usual activities, despite some symptoms.
2: slight disability – able to look after own affairs without assistance, but unable to carry out all previous activities.
3: moderate disability—requires some help, but can walk unassisted
4: moderately severe disability—unable to attend to own bodily needs without assistance, and unable to walk unassisted.
5: severe disability—requires constant nursing care and attention, bedridden, incontinent.
6: dead.

We defined mRS of 0–2 as desirable recovery level (dRL) and mRS of 3 to 6 as non-dRL referred to related research. Since dRL was easily communicable, understandable, and desirable to patients and neurosurgeons, it was chosen as a primary study endpoint of our study.

### Data Collection and Analysis

The following CSF dynamics parameters were extracted and calculated from our database: ICP baseline (ICPb), ICP at plateau (ICPp), cranioplasty date (if applicable), resistance to outflow (Rout), and the correlation coefficient between the ICP wave amplitude and the mean ICP level (RAP), which is also called the compensatory reserve index. In our study, to avoid the inaccuracy of instant RAP value in a prognosis, we also designed a maximum RAP value as RAPmax, which stands for the highest RAP parameter during different date segments (RAP1 max represents the baseline RAPmax at the beginning of continuous lumbar drainage, RAP2max represents the highest RAP parameter from the start to the 1st day end, and RAP3 max represents the highest RAP parameter for the 2nd day), and defined ΔRAPmax as (baseline RAPmax – interim RAPmax)/baseline RAPmax × 100 (%). Additional clinical data: patient demographics, date/severity of TBI, date of infusion test, continuous lumbar drainage study, and brain imaging were extracted from the electronic hospital record system.

IBM SPSS 22.0 (IBM Corp. New York, USA) and MedCalc 19.0 (MedCalcSoftware bvba, Ostend, Belgium) were used for statistical analyses. Data were described as numbers (percentages) or means ± standard deviation (SD). The predictive performance of parameters was tested by receiver-operating characteristic (ROC) curve analysis. The logistic regression model was used to evaluate different predictive parameters and calculated predictive probability used for ROC analysis. Optimal cut-off values to predict shunting response were calculated by the Youden index using MedCalc19.0 software. We have used Pearson's or Spearman's correlation when appropriate, depending on how much our data deviated from a bivariate normal distribution or an asymptotically normal distribution, and P-values of less than 0.05 were considered statistically significant.

## Results

### Patients' Characteristics in Training Set

Table 2 summarizes the characteristics of patients in the training set. About 191 patients with PTH with Rout and the dRL 3 months after shunting were retrospectively analyzed, among whom 134 patients (70.2%) were treated with VP shunting based on clinical and radiological assessment. From the records, 134 (70.1%) had been initially classified as having “severe” and 57 (29.9%) “mild” TBI according to the Glasgow Coma Scale (GCS). The interval time between TBI and infusion test varied between subjects, from 2 weeks to 18 weeks; average time, 8.2 ± 3.1 weeks. Of patients, 50.3% (96/191) achieved dRL 3 months later.

**Table 2:** Baseline characteristics of patients in the training set and the validation set.

**Characteristics**	**Patients**	
	**Training set** **(*n* = 191)**	**Validation set** **(*n* = 70)**
Age (mean ± SD, years)	46.3 ± 8.7	48.5 ± 6.9
GCS		
>8 (mild)	57 (29.9%)	46 (65.7%)
≤ 8 (severe)	134 (70.1%)	24 (34.3%)
Decompressive craniotomy	36 (18.8%)	11 (15.7%)
Interval time between TBI and infusion (*x* ± s, weeks)	8.2 ± 3.1	7.4 ± 2.8
Shunting strategy		
VP shunting	134 (70.2%)	51 (72.9%)
LP shunting	57 (29.8%)	19 (27.1%)
ICPb (mean ± SD, mmHg)	10.3 ± 4.3	11.3 ± 3.3
ICPdra (mean ± SD, mmHg)	8.9 ± 3.4	9.13 ± 2.9
Rout ($\bar{x}\pm s$, mmHg/min/ml)	14.1 ± 3.3	13.7 ± 4.1
RAPb (mean ± SD)	/	0.70 ± 0.14
RAPdra (mean ± SD)	/	0.57 ± 0.13
RAPmax		
RAP1max	/	0.70 ± 0.14
RAP2max	/	0.57 ± 0.10
RAP3max	/	0.34 ± 0.13
The Modified Rankin Scale		
dRL	96 (50.3%)	38 (54.4%)
Non-dRL	95 (49.7%)	32 (45.6%)

*GCS, Glasgow coma scale; LP, lumboperitoneal; VP, ventriculoperitoneal; ICPb, intracranial pressure at the baseline; ICPdra, intracranial pressure during drainage; Rout, resistance to outflow; RAPb, compensatory reserve index (the correlation coefficient between the ICP wave amplitude and the mean ICP level) at the baseline; RAPdra, RAP during drainage; RAPmax, a maximum RAP; dRL, desirable recovery level (the modified Rankin Scale of 0 to 2); GCS, Glasgow coma scale; LP, lumboperitoneal; VP, ventriculoperitoneal; ICPb, intracranial pressure at the baseline; ICPdra, intracranial pressure during drainage; Rout, resistance to outflow; RAPb, compensatory reserve index (the correlation coefficient between the ICP wave amplitude and the mean ICP level) at the baseline; RAPdra, RAP during drainage; RAPmax, a maximum RAP; dRL, desirable recovery level (the modified Rankin Scale of 0 to 2)*.

### Patients' Characteristics in the Validation Set

From October 2018 and February 2020, 82 patients were enrolled in our study, and underwent CT scan and Rout assessment, but 12 of them were excluded because of bad physiological conditions, which could not get them through shunting (*n* = 8) and withdrawal consent with the reason of being short of money or other individual subjective reasons (*n* = 4). Finally, 70 patients with evaluable Rout, RAP, and outcome data were eventually included for statistical analysis. All patients with PTH were treated with shunting. Of them, 72.9% (51/70) received VP shunting, 27.1% (19/70) received LP shunting. The follow-up time for evaluation based on the mRS was designed at 3 months after shunting, but, finally, 70% (47/70) of them accomplished the follow-up at 3 months, 8.6% (6/70) and 7.1% (5/70) fulfilled ≥ 4 months and 5 months, respectively, due to patients' personal causes. Overall, dRL was achieved in 54.4% of the patients (38/70).

### Rout Predicts dRL in Training Set

**Figure 1:**
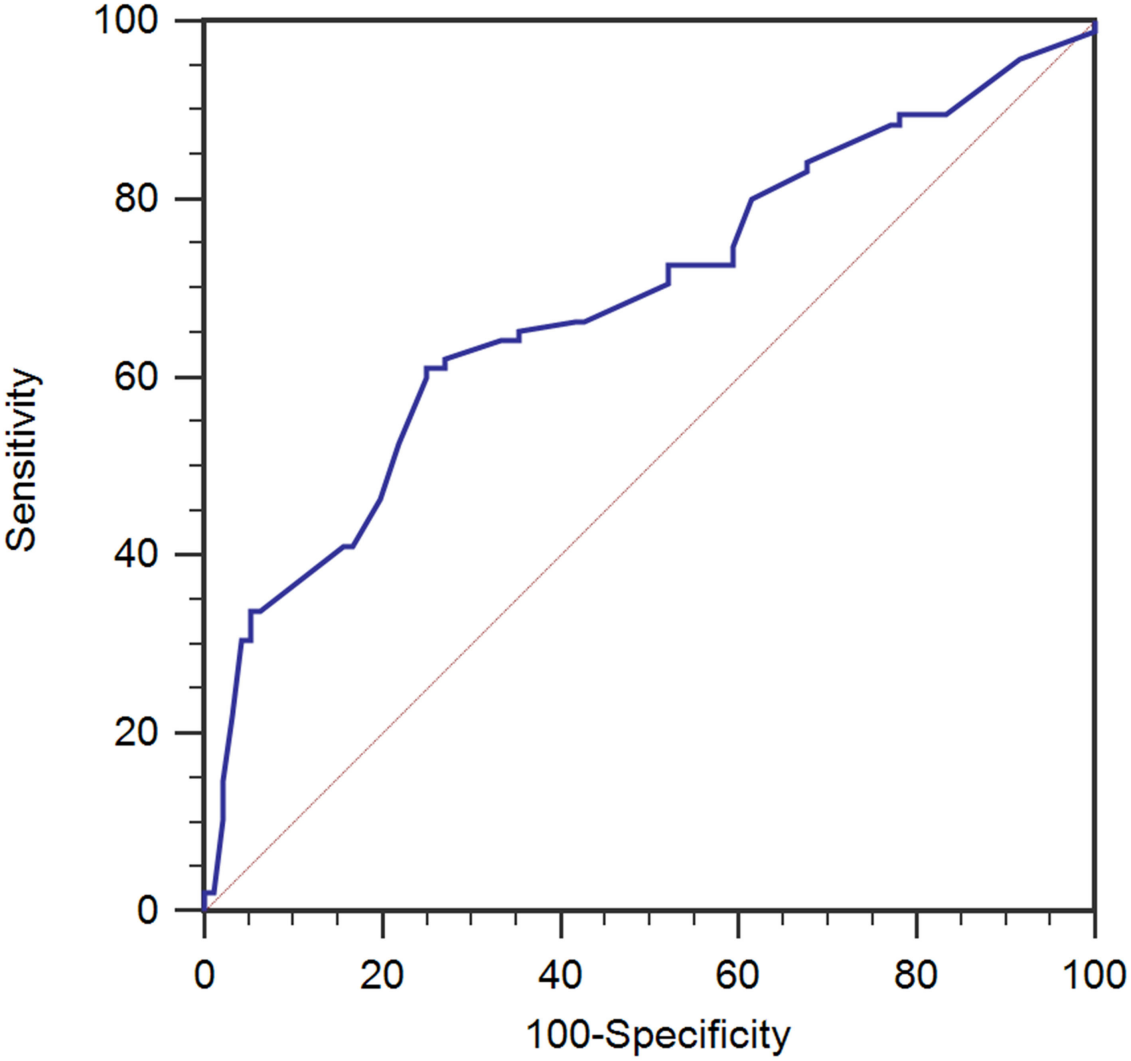
ROC analysis for therapeutic CSF outflow (Rout) in the prediction of desirable recovery level (dRL) in the training set (*n* = 191). Rout was a predictive factor in dRL with the area under the curve (AUC) of 0.686 [95% confidence interval (CI) 0.615–0.751, *p* < 0.001]. ROC, receiver-operating characteristic.

In the training set, Rout was 14.1 ± 3.3 mmHg/min/ml (mean ± SD). We used the ROC curve to assess the value of Rout in predicting the dRL to shunting independently (Figure 1), and then acquired an AUC of 0.686 (95% confidence interval [CI] 0.615–0.751, *p* < 0.001). It suggested that pretherapeutic Rout was a dRL predictor of shunting in patients with PTH. Cut-off value of 13.6 for Rout offered the best accuracy in predicting dRL with the sensitivity of 61.05%, specificity of 75.00%, positive predictive value (PPV) of 70.7%, and negative predictive value (NPV) of 66.1%.

### Rout and ΔRAPmax% Predict dRL in the Validation Set

**Figure 2:**
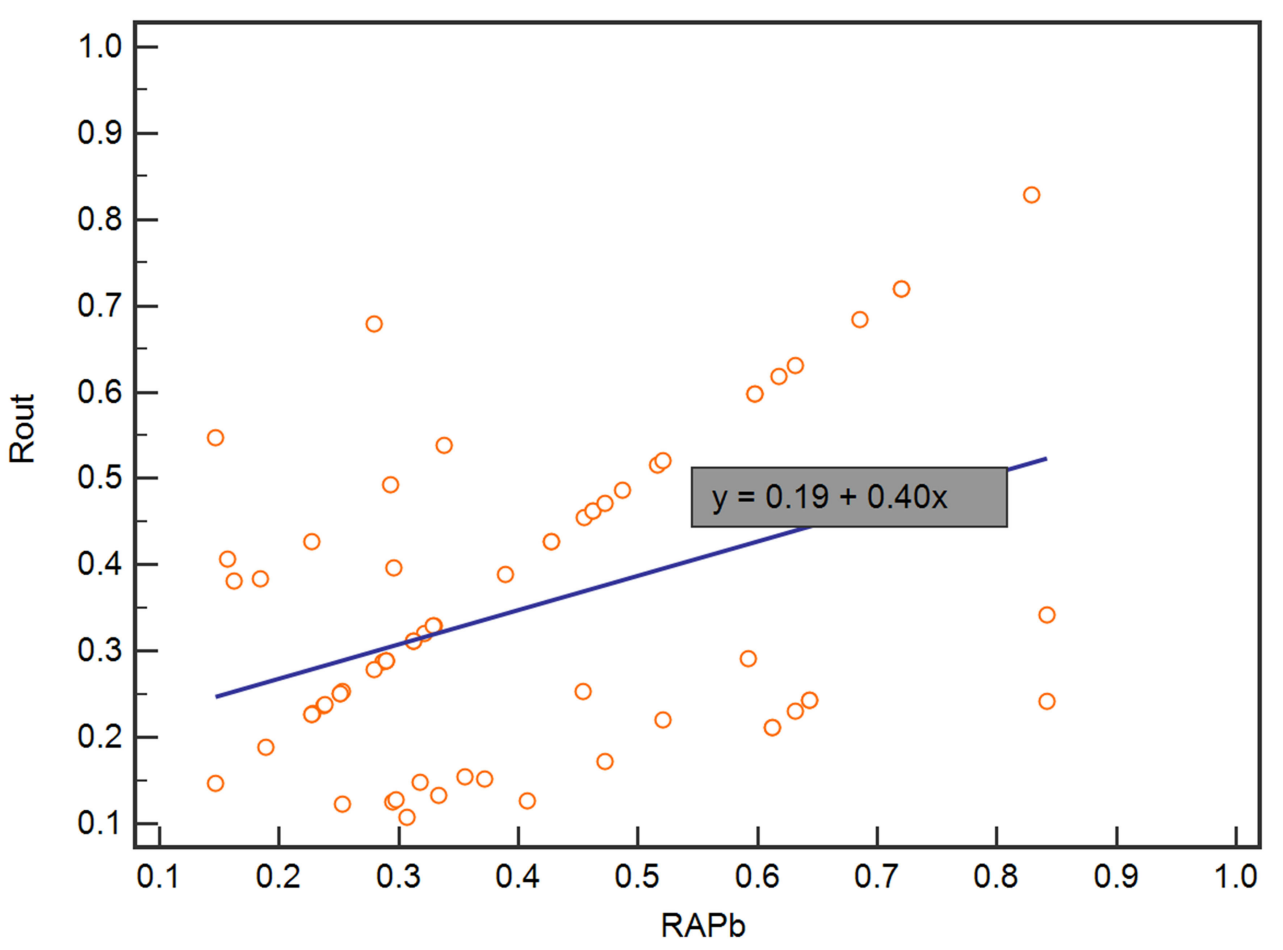
The scatterplot showed the correlation between RAPb and Rout. RAPb and Rout were significantly correlated with a moderate coefficient of 0.40. RAPb, RAP at the baseline.

**Figure 3:**
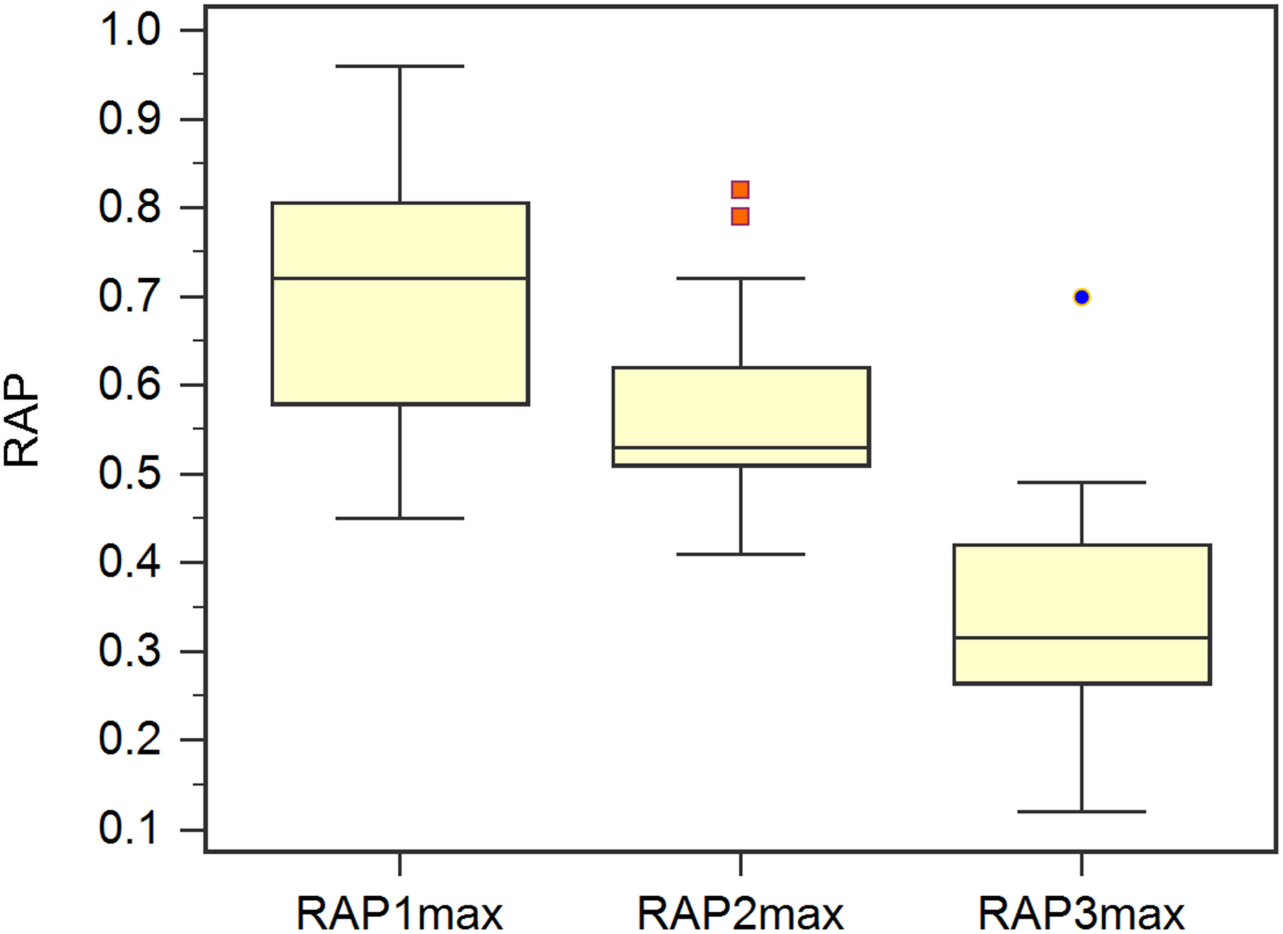
The scatterplot showed the correlation between ΔRAP1max% and ΔRAP2max%. ΔRAP1max% and ΔRAP2max% were significantly correlated with a coefficient of 0.59. RAPmax, maximum RAP.

**Figure 4:**
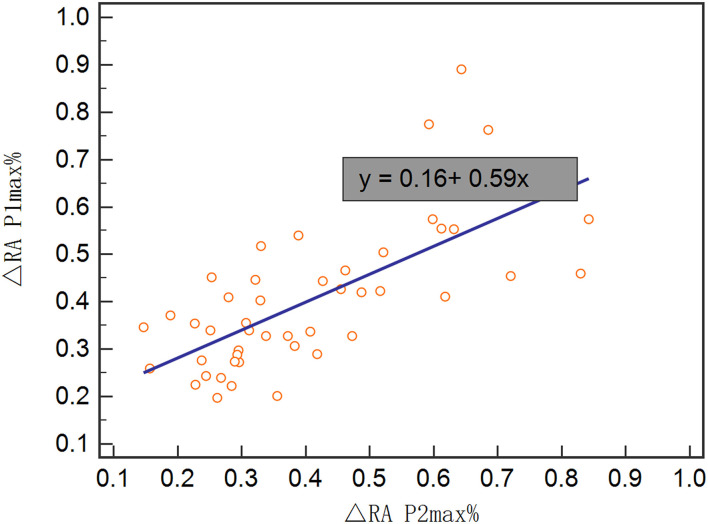
The box plot showed the maximum RAP (RAPmax) of the baseline (RAP1max), post 1 and 2 days (RAP2max and RAP3max).

**Figure 5:**
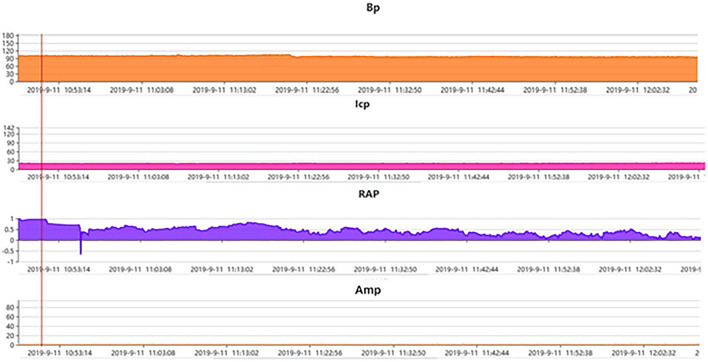
A representative example of pressure-volume change in a patient with posttraumatic hydrocephalus. Post traumatic hydrocephalus. ICP at the baseline 12–15 mmHg (monitored *via* lumbar puncture in this case) and Amp at the baseline ~3 mmHg both stayed steady after the start of continuous lumbar drainage, while RAP at the baseline ~1. clearly decreased to almost 0.6 after drainage of only a few minutes and kept the declining trend in the following hours, indicating the recovery of the compensatory reserve. AMP, fundamental amplitude of ICP. RAP, compensatory reserve index (moving correlation coefficient between ICP and AMP).

In the validation set, Rout was 13.7 ± 4.1 mmHg/min/ml. Then, all 70 patients were served with continuous lumbar drainage tests to collect RAP at the baseline (RAPb) and during drainage (RAPdra). The correlation between Rout and RAPb was significant with a moderate correlation coefficient of 0.40 (*p* < 0.001; Figure 2). Baseline RAPmax (RAP1max, mean ± SD) was 0.70 ± 0.14, the 1st-day end and the 2nd-day end of continuous lumbar drainage, mean RAPmax's (RAP2max and RAP3max) were 0.57 ± 0.10 and 0.34 ± 0.13, respectively (Figure 3). MeanΔRAPmax's% after 1 and 2 days (ΔRAP1max% and ΔRAP2max %) were 40.2 ± 15.1% and 40.4 ± 17.4%, respectively. The correlation coefficients between dRL and ΔRAP1max %, dRL, and ΔRAP2max % were 0.67 (*p* < 0.001) and 0.69 (*p* < 0.001), respectively. The correlation between ΔRAP1max% and ΔRAP2max% was significant with a correlation coefficient of 0.59 (*p* < 0.001; Figure 4). A representative example of a continuous lumbar drainage test performed on a patient with posttraumatic hydrocephalus is shown in Figure 5.

Firstly, we performed univariate logical regression with all parameters. Then, significance threshold of *p* < 0.10 removed age, shunting strategy, ICPb, ICPdra, RAPb, RAPdra from the comparison. And put the remaining parameters, including Rout (*p* = 0.06), ΔRAP1max% (*p* < 0.001), andΔRAP2max% (*p* < 0.001), into multivariate logistic regression to conduct multiple regression models. Finally, 5 separate regression models (Table 3) were analyzed for their ability to predict the shunting response of patients with PTH. The predictive model of the total index shows the largest AUC = 0.88 and highest significance of the Hosmer–Lemeshow test (0.211) among the 5 models, indicating its better performance in the goodness of fit.

**Table 3:** Rout, ΔRAP1max%,ΔRAP2max% and combined index models for measurement results in the validation set.

	**AUC**	**Sensitivity**	**Specificity**	**Hosmer-Lemeshow**
Rout	0.634 (0.51–0.75)	42.11 (26.30–59.20)	84.37 (67.20–94.70)	0.072
ΔRAP1max%	0.773 (0.66–0.86)	89.47 (75.20–97.10)	56.25 (37.70- 73.60)	0.132
ΔRAP2max%	0.786 (0.67–0.88)	76.32 (59.80–88.60)	75.00 (56.60–88.50)	0.103
ΔRAP1max% and ΔRAP2max%	0.804 (0.76–0.93)	76.32 (59.80–88.60)	90.12 (75.01–98.03)	0.140
Total index	0.879 (0.78–0.95)	81.58 (65.70–92.30)	90.62 (75.00–98.00)	0.211

*The predictive model of total index shows the largest AUC = 0.88 and highest significance of Hosmer-Lemeshow test (0.211) among the 5 models, indicating its better performance in goodness of fit. However, excluding Rout from the total index model lowered its effectiveness to AUC of 0.81 and significance of Hosmer–Lemeshow test of 0.140. Values are shown with [95% CI]. AUC, area under the receiver-operator Curve; total index means the combination model of Rout, ΔRAP1max%,ΔRAP2max%*.

**Figure 6:**
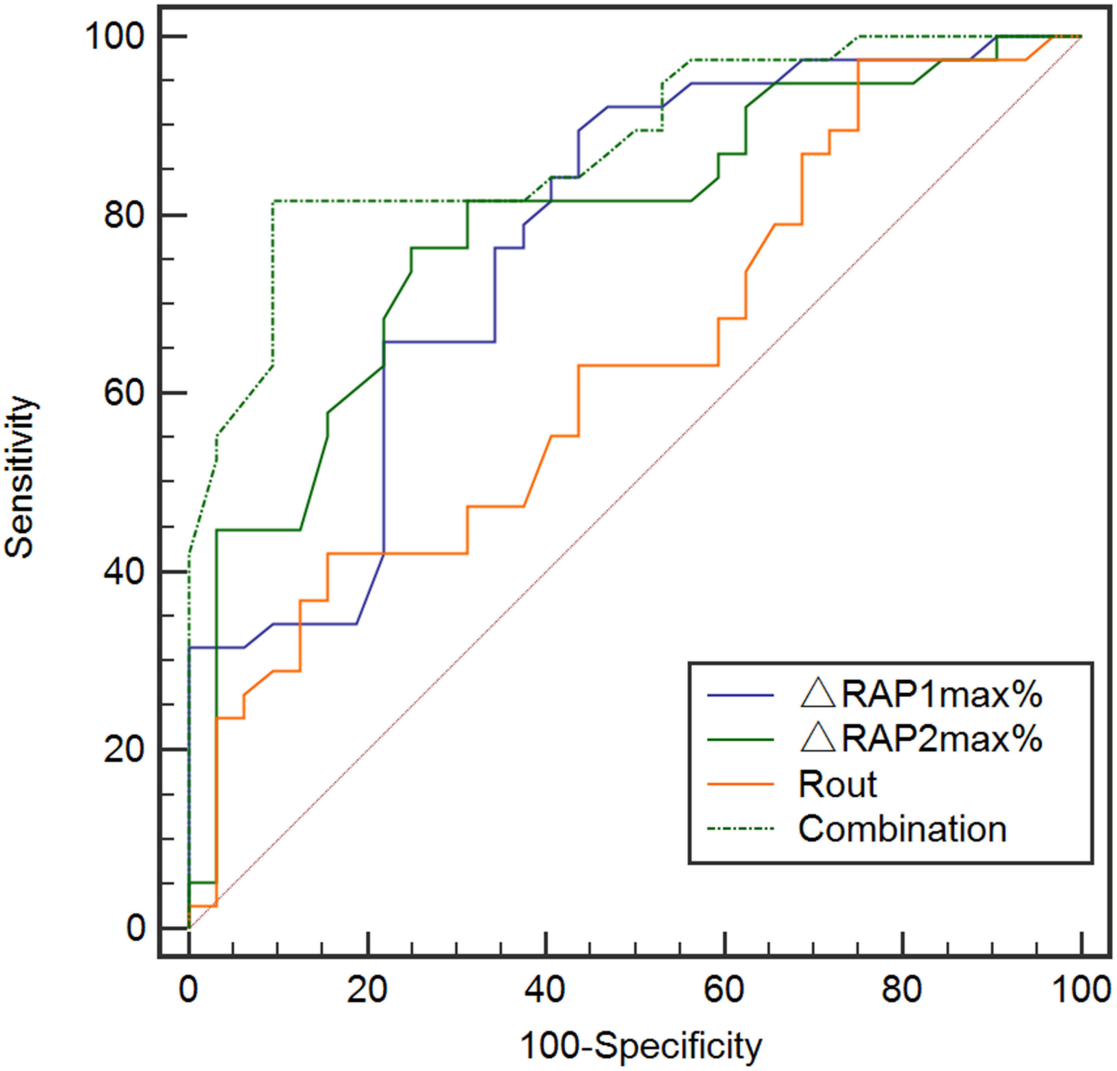
ROC analysis for Rout, ΔRAP1max%, ΔRAP2max%, and the overall combination of them in the prediction of dRL in the validation set (*n* = 70). Rout alone did not show significant value in predicting dRL with the AUC of 0.634 (95% CI, 0.511–0.746, *p* = 0.0613). But either ΔRAP1max% or ΔRAP2max% could effectively predict dRL, with the AUC of 0.773 (95% CI, 0.657–0.864, *p* < 0.001) and 0.786 (95% CI, 0.671–0.875, *p* < 0.001), respectively. ROC analysis with combination of Rout, ΔRAP1max%, and ΔRAP2max% in prediction dRL in the validation set. The combination of Rout and ΔRAPmax% maximally increased the predictive power with the largest AUC of 0.879 (95% CI, 0.671–0.875, *p* < 0.001). AUC, area under the curve; CI, confidence interval; dRL, desirable recovery level (the modified Rankin Scale of 0 to 2); ROC, receiver-operating characteristic; RAPmax, maximum standardized uptake value.

Consequently, we evaluated the predictive value of Rout, ΔRAP1max%, and ΔRAP2max% alone using ROC analysis in the validation set (Figure 6). There was no significant value for Rout in prediction dRL with the AUC of 0.634 (95% CI, 0.511–0.746, *p* = 0.0613). However, either ΔRAP1max% or ΔRAP2max% could effectively predict the dRL of shunting in the primary stage of PTH, with the AUC of 0.773 (95% CI, 0.657–0.864, *p* < 0.001) and 0.786 (95% CI, 0.671–0.875, *p* < 0.001), respectively. At a cut-off of 45%, ΔRAP1max% offered the best accuracy in predicting dRL with the sensitivity, specificity, PPV, and NPV of 89.5%, 56.3%, 70.8%, and 81.8%, respectively; 53% was the optimum cut-off of ΔRAP2max%, and it discriminates between dRL and non-dRL. The sensitivity, specificity, PPV, and NPV were 76.32%, 75.00%, 78.4%, and 72.7%, respectively.

To exploit the predictive value of association of Rout and ΔRAPmax%, we united Rout with ΔRAP1max % and ΔRAP2max % in the logistic analysis model, and the predictive model of total index shows the largest AUC = 0.88 (95% CI, 0.671–0.875, *p* < 0.001) and highest significance of Hosmer–Lemeshow test (0.211) among the 5 models, indicating its better performance in the goodness of fit (Figure 6). The sensitivity, specificity, PPV, and NPV of the total index model to predict dRL were 81.62%, 90.62%, 91.2%, and 80.6%, respectively.

### RAPmax Predicts Non-dRL (Levels 3 to 6 in Modified Rankin Scale)

**Figure 7:**
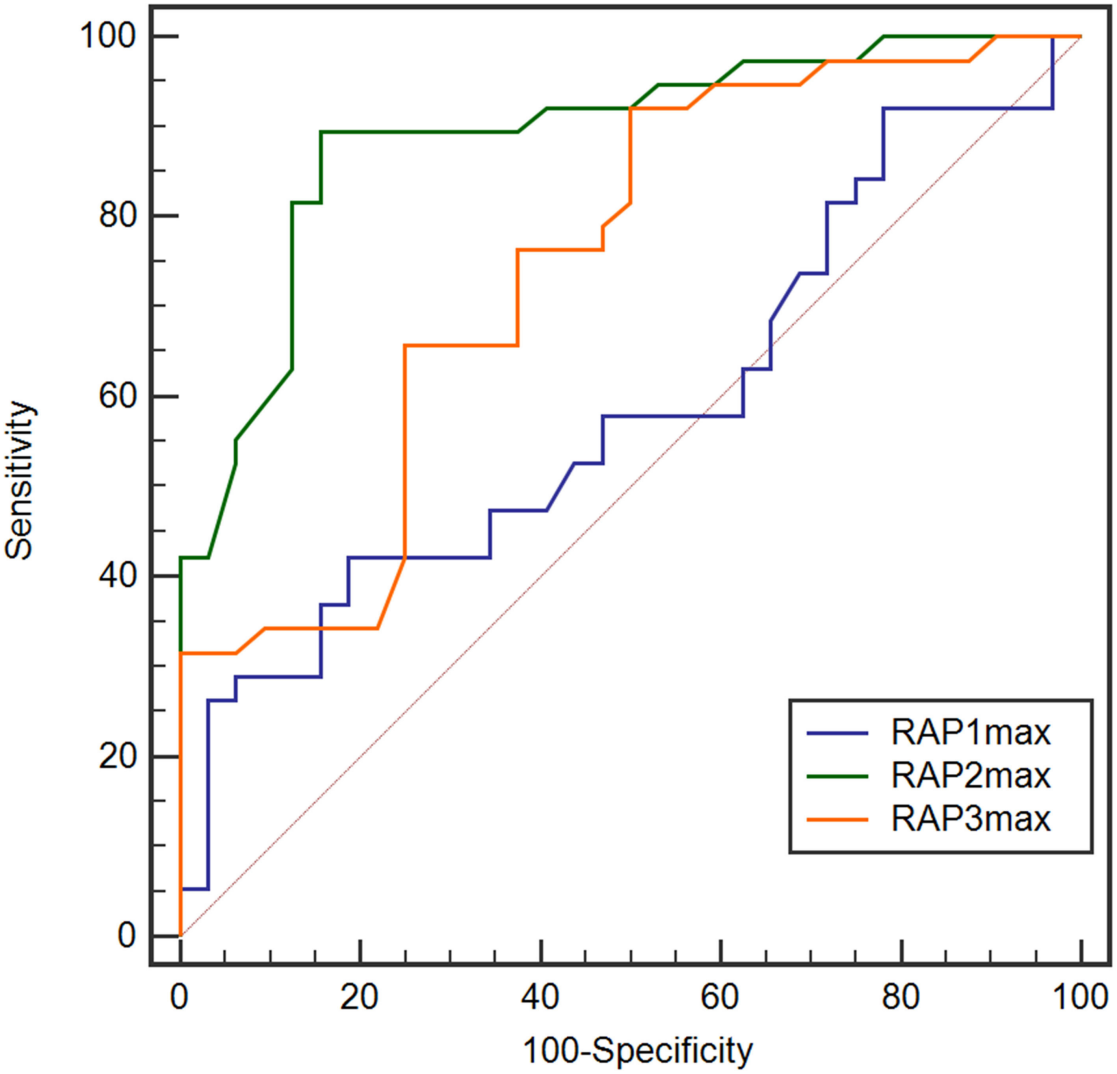
ROC analysis for RAP1max, RAP2max, and RAP3max in the prediction of non desirable recovery level (non-dRL). RAP2max and RAP3max showed remarkable value in prediction of non-dRL with the AUC of 0.891 (95% CI, 0.793–0.953, *p* < 0.001) and 0.746 (95% CI, 0.628–0.842, *p* < 0.001), but RAP1max still showed no predictive value (AUC of 0.587, *p* = 0.2058). AUC, area under the curve; CI, confidence interval; non-dRL, nondesirable recovery level (the modified Rankin Scale of 3 to 6); ROC, receiver-operating characteristic; RAPmax, maximum RAP.

We also explored the predictive value of RAPmax and found the baseline RAPmax (RAP1max), post-1-day RAPmax (RAP2max) and post-2-day RAPmax (RAP3max) all could not predict dRL (figures were not shown in the article). However, when we changed the target parameter as totally non-dRL (Levels 3 to 6 in modified Rankin Scale), the RAP2max and RAP3max revealed remarkable predictive value with the AUC of 0.891 (95% CI, 0.793–0.953, *p* < 0.001) and 0.746 (95% CI, 0.628–0.842, *p* < 0.001; Figure 7), RAP1max still showed no predictive value (AUC of 0.587, *p* = 0.2058). With the cut-off value at 0.65 of RAP2max, the sensitivity, specificity, PPV, and NPV to predict the non-dRL were 89.5%, 84.4%, 67.2%, and 97.1%, respectively. In addition, 0.51 was the optimum cut-off for RAP3max in predicting the non-dRL with the sensitivity, specificity, PPV, and NPV of 92.11%, 50.00%, 58.6%, and 94.2%, respectively.

In summary, our correlation analysis subsequently revealed a positive correlation between Rout and RAP at the baseline (r = 0.40, *p* < 0.001) (Figure 2). It indicated that, in patients with higher Rout (suggesting abnormal CSF circulation), the accompanied RAP at the baseline may often reach a higher level approaching 1 (meaning poorer compensatory reserve). Then, we originally hypothesized that the model of Rout united with RAPb could present excellent ability in predicting a prognosis. Unfortunately, in our univariate logical regression, neither RAPb (*p* = 0.231) nor RAPdra (*p* = 0.304) showed significance in evaluating the outcome of patients with PTH after shunting. However, the RAP-related parameters, ΔRAP1max (<0.001) and ΔRAP2max (*p* < 0.001), which weighed the maximum variation of RAP value based on RAPb; both entered the multivariate logistic regression. There was also a strong association between dRL and ΔRAP1max% with a correlation coefficient of 0.67 (*p* < 0.001), dRL and ΔRAP2max% with 0.69 (*p* < 0.001). We further combined Rout with ΔRAP1max% and ΔRAP2max%; the AUC in predicting dRL reached 0.879 (*p* < 0.001). However, excluding Rout from the total index model lowered its effectiveness to an AUC of 0.81.

Therefore, ΔRAP1max% should be taken more attention in clinical observation. Moreover, we found ΔRAP2max% was significantly correlated with ΔRAP1max% when RAPmax during the 1st day had a significant decrease; the decrease would last to the 2nd day. It suggested that the continuous drainage, which imitated shunting, continued to be effective in some patients. Therefore, the ΔRAP1max% after the drainage of the 1st day was efficient to predict the PTH patient's dRL.

### Complications

There were no complications or side effects related to the lumbar infusion test or continuous lumbar drainage test. The shunting procedure was complicated by a subdural hematoma in two patients: in one of these, the hematoma was solved surgically; in the other one, the hematomas did not require dissipated and were absorbed spontaneously. One patient had a postoperative shunt infection with encephalitis and was treated with antibiotics and removal of the shunt device. After 2 months, a new shunt was implanted. All complications had been resolved before the follow-up assessment.

## Discussion

We found that pre-shuntingRout in primary PTH could predict the outcome of shunting based on the modified Rankin Scale (mRS) in the training set. And in the validation set, the relative change of maximum RAP value in the early phase of PTH can make a prediction for dRL (an mRS running from 0 to 2) to shunting in advance. However, Rout in the validation set did not show significant value in forecasting dRL to shunting. But the combination of Rout and ΔRAPmax% maximally increased the competence of prediction. The RAPmax value at the baseline and post 1 day or post 2 days of the drainage did not reveal a significant predictive value for dRL, but the latter 2 parameters were found to be strongly independent predictors for non-dRL (an mRS running from 3 to 6) response.

Post-acute PTH could block the rehabilitation of brain physiology and be the main contributing factor in poor long-term outcomes after TBI (28, 29). Even in the late phase after TBI, patients can emerge symptoms or signs resembling idiopathic normal pressure hydrocephalus (iNPH) due to impairment of CSF circulation in the subarachnoid space in response to the posttraumatic inflammatory process (30, 31). Measurements of opening pressure, such as baseline ICP and CSF dynamics, *via* lumbar puncture and spinal tap test, are often used to investigate PTH and screen outpatients for shunting, but fall of diagnostical accuracy (23, 32) since Rout depending on its CSF dynamic characteristic, designed by Marmarou (33, 34), was widely used to assess the indication for shunting in a lumbar infusion test, which was first presented by Katzman et al. (35) and improved by Czosnyka et al. (36). However, the thresholds of Rout for shunting varied from iNPH and NPH-related pieces of research, and it has not been explored sufficiently in PTH cohorts. In our study, we firstly evaluated Rout to serve as a predictor for dRL using ROC analysis in the training set and acquired a slightly low but statistically significant AUC of 0.686 (*p* < 0.001), mean of 14.1 mmHg/min/ml, and best cut-off value of 13.6, which were close to the conventional Rout threshold of 13 mmHg/min/ml (10, 16). This indicated that Rout was a predictor for dRL, which is consistent with previous research, but its predictive value was limited with a relatively low AUC. Therefore, Rout should be combined with more parameters to enhance its predictive power.

Our study confirmed the predictive value of Rout collected from the Infusion test and RAPmax% derived from the initial RAP of continuous drainage prospectively. Unfortunately, we did not reach a statistically significant value in Rout predicting dRL (AUC of 0.634, *p* = 0.0613) in the validation set probably. This could be interpreted by confounding factors mixing in single-factor analysis without recognition or limitation in insufficient sample size. To avoid some cases, the meaningful influence factors lose the opportunity to enter a multivariate model. Firstly, we broadened the significance threshold to *p* < 0.10 when performing univariate logical regression with all parameters. These significant parameters, including Rout (*p* = 0.06), ΔRAP1max% (*p* < 0.001), and ΔRAP2max% (*p* < 0.001), were selected out and entered the multivariate logistic regression to conduct multiple regression models. Finally, 5 separate regression models (Table 3) were analyzed for their ability to predict the shunting response of patients with PTH. Since some previous investigations suggested that Rout showed a negative correlation with auto-regulation in patients with NPH (11, 37), RAP as an effective compensatory reserve index may present a potential connection with Rout in the pretherapy phase. Our results suggested that Rout assessed routinely in pre-shunting could additionally enhance the value of predicting dRL of patients with PTH after shunting. Although ΔRAP2max% seemed to reach a better predictive value with AUC of 0.786 (*p* < 0.001), compared with ΔRAP1max% with AUC of 0.773 (*p* < 0.001), actually, initial parameter reflecting the responsiveness of shunting is more valuable as a guide to subsequent treatment.

Many previous researchers have explored the value of RAP in early evaluating the efficacy of an outcome in hydrocephalus, depending on its remarkable performance in indicating when the equilibrium of CSF is disturbed or pressure-volume compensation is poor (18, 19, 38). But there was still no unanimously agreed threshold of RAP for guiding the surgical time of shunting. Schuhmann et al. found that there was a transition point, suggesting 0.6 of RAP, below which the relationship between CSF pressure and volume is linear, and the whole system is considered stable, while above which the compensatory reserve ability of brain began to be damaged and the equilibrium of CSF was disturbed, too (11). However, this transition point was difficult to determine clinically and varied overtly in patients with different causes of hydrocephalus. Even in the current study, RAP, both at the baseline and at the plateau, showed no significant value in evaluating PTH (22). In our study, we firstly discovered the cut-off values of ΔRAPmax%, and the 45% cut-off of ΔRAP1max% and the 53% cut-off of ΔRAP2max% both provided great performance to predict dRL. Because this parameter mainly emphasizes the variation of overall RAP during the specific monitoring period, it may be better to assess the outcome of patients with PTH after shunting. But a further prospective large-scale study is necessary to verify this point.

There were a majority of patients with PTH suffered from spinal stenosis and deformity after TBI. Besides, the shunt tubes in LP shunting were more easily blocked compared with VP shunting. These reasons led to unbalanced distribution on shunting strategies. However, in our study, the shunting strategy did not show a significant difference in predicting the prognosis (*p* = 0.541). In a related study, LP shunt surgery was equally as effective as the VP shunt surgery in the treatment of moderate and severe coma patients with posthemorrhagic communicating hydrocephalus (39).

There were some limitations in our study. We enrolled some heterogeneous groups of patients with TBI with different types of injuries or surgical operations, such as decompressive craniotomy (DC) and non-DC, but we were not able to perform a further detailed analysis of in an inter-group due to limited small sample size. Furthermore, the time frame post-TBI varied from weeks to months, and, in some cases, the exact date of the TBI or cranioplasty was not available. Because the time delay of untreated chronic hydrocephalus impacts the possibility of improvement post-shunting, the heterogeneity of our sample may have affected our ability to interpret improvement post-shunting in patients with PTH. In addition, the outcome of the follow-up based on the modified Ranking Scale may also have some bias, owing to characteristics of patients with brain trauma. It means that patients with TBI may benefit well from the shunting in terms of hydrocephalus. However, they could not achieve a suitable score on mRS as a result of paralysis caused by TBI. It is hard to differentiate dyskinesia from hydrocephalus and posttraumatic brain syndrome only through mRS. A prospective clinical study with larger sample size better with a multi-center to prove the predictive value of Rout, ΔRAPmax%, and their combination in different subtypes of PTH is needed.

## Conclusion

Either RAP-related parameters or Rout can be used for early prediction of dRL to shunting on patients with PTH. ΔRAPmax% combined Rout could be used as a better predictor of shunting response for patients with PTH. This may help the neurosurgeons predict the probability of achieving dRL and facilitate optimization of the individual treatment plan by avoiding ineffective and unnecessary surgery.

## Data Availability Statement

The original contributions presented in the study are included in the article/supplementary material, further inquiries can be directed to the corresponding author/s.

## Ethics Statement

The studies involving human participants were reviewed and approved by Clinical Research Ethics Committee of The First Affiliated Hospital, Zhejiang University School of Medicine. The patients/participants provided their written informed consent to participate in this study.

## Author Contributions

All authors listed have made a substantial, direct, and intellectual contribution to the work and approved it for publication.

## Funding

This study was supported by the National Nature Science Foundation of China (Grant No. 81971699) and the Clinical Research Innovation Plan of Shanghai General Hospital.

## Conflict of Interest

The authors declare that the research was conducted in the absence of any commercial or financial relationships that could be construed as a potential conflict of interest.

## Publisher's Note

All claims expressed in this article are solely those of the authors and do not necessarily represent those of their affiliated organizations, or those of the publisher, the editors and the reviewers. Any product that may be evaluated in this article, or claim that may be made by its manufacturer, is not guaranteed or endorsed by the publisher.
